# Growth Stunting and Nutritional Deficiencies among Children and Adolescents with Celiac Disease in Kuwait: A Case–Control Study

**DOI:** 10.3390/children11091042

**Published:** 2024-08-27

**Authors:** Esraa Almahmoud, Dalal Usamah Zaid Alkazemi, Wafaa Al-Qabandi

**Affiliations:** 1 Department of Food Science and Nutrition, College of Life Sciences, Kuwait University, P.O. Box 17438, Al-Shadadiya 72455, Kuwait; dietitian.e@gmail.com; 2 Department of Pediatrics, Faculty of Medicine, Kuwait University, P.O. Box 24923, Jabriya 90805, Kuwait; wafaa.alqabandi@ku.edu.kw

**Keywords:** celiac disease, children, adolescents, nutritional status, growth stunting, wasting, vitamins, failure to thrive, gluten-free diet

## Abstract

Background/Objectives: This study aimed to assess the nutritional status of children and adolescents with celiac disease (CD) in Kuwait and investigate the nutritional deficiencies and sociodemographic factors associated with growth stunting in this population. Methods: This case–control study included 77 CD patients aged 3–18 years diagnosed with CD using IgA anti-tissue transglutaminase and duodenal biopsy and 33 healthy controls. Nutritional status was evaluated based on demographic and clinical characteristics, anthropometric measurements, and biochemical parameters. Univariate and multivariate logistic regression models were used to determine the association between CD and growth stunting. Results: Approximately one-third (31%) of children with CD had stunted growth, 20.8% had a low body mass index for their age, and 5.2% had both growth stunting and wasting. Children with CD had higher odds of iron-deficiency anemia, vitamin D deficiency, anemia, and lower socioeconomic status. They were also younger and had decreased serum levels of vitamin D compared to the controls. These factors were all significantly associated with an increased risk of CD, collectively explaining over 50% of the risk. For growth stunting, lower education status among mothers, family income, and serum ferritin were identified as risk factors. Conclusions: A significant proportion of children and adolescents with CD had malnutrition, overt deficiencies, and impaired growth despite coherence with a gluten-free diet. Recommendation: Routine monitoring and targeted nutritional interventions are recommended for children and adolescents with CD to address malnutrition and growth stunting. Addressing socioeconomic disparities and enhancing maternal education may also help mitigate the risk factors.

## 1. Introduction

Celiac disease (CD) is an immune-mediated enteropathy characterized by chronic bowel inflammation with severe adverse consequences on children’s health and development [1]. Patients with CD present with variable clinical features, with its typical presentation mainly occurring in the first 2 years of life after the introduction of gluten into the child’s diet [2]. The disease’s impact extends beyond the gastrointestinal tract, affecting growth, development, and overall health.

As enteropathy develops, clinical symptoms of malabsorption appear, such as diarrhea, failure to thrive (FTT), abdominal distension, pain, and iron-deficiency anemia (IDA) [3]. Early symptoms may include poor weight gain, growth retardation, and delayed puberty due to inadequate nutrition or malabsorption [4]. Additionally, children with atypical symptoms of CD are often overweight and obese, which also negatively influences their growth and health status [5].

In Kuwait, Al-Qabandi et al. found that the most reported symptoms of CD were FTT (72%), diarrhea (64%), and abdominal distension (56%) [6]. Regarding the weight status of patients with CD, 19% were underweight, 17% were overweight, and 14% were obese [6]. This variation in weight status indicates a complex interplay between CD and nutritional outcomes that warrants further investigation.

FTT or impaired growth has long been recognized as a sign of CD. However, in Kuwait, the factors associated with growth failure among patients with CD remain unknown. Proposed mechanisms include histological damage leading to nutrients’ malabsorption, abnormalities in growth hormone (GH) and insulin-like growth factor 1 (IGF-1) levels, and increased levels of anti-pituitary antibodies [7]. Significant improvement in growth occurs when patients follow a gluten-free diet (GFD); however, reduced adult height remains a possibility [8]. Therefore, understanding the nutrition-related factors associated with poor growth in patients with CD is crucial to preventing compromised growth and ensuring better health outcomes.

Children with CD require adherence to a GFD, which can lead to nutritional deficiencies. Studies have shown varying prevalence of deficiencies among nutrients in children following a GFD, with iron, ferritin, and vitamin D deficiencies being the most common [9]. Despite proactive follow-up protocols, adherence to long-term monitoring is poor, with only 39% of patients continuing follow-up five years post-diagnosis [10]. However, a study of Saudi children following a Ministry of Health GFD program showed generally good nutritional status, with normal anthropometric indices and biochemical parameters, except for vitamin D levels, which were below the normal range [11]. Factors influencing nutritional status included age, family income, and family size [11]. These findings highlight the importance of structured nutritional assessment and monitoring in pediatric CD patients to prevent and address potential deficiencies while following a GFD.

Evaluating the nutritional status of children with CD offers a comprehensive approach to developing population-specific nutritional recommendations and reducing the risk of poor health and malnutrition. Globally, several studies have highlighted the importance of nutritional monitoring and intervention in CD patients to improve their growth and health outcomes [11,12,13]. A GFD is the primary treatment for CD, but it can lead to nutritional deficiencies and metabolic complications [14,15]. Regular nutritional assessment and monitoring are essential for patients with CD, both at diagnosis and during long-term GFD adherence [16,17]. New tools for monitoring GFD adherence and assessing nutritional status are emerging, which could enhance follow-up strategies [18]. Registered dietitian nutritionists play a crucial role in educating patients about the GFD, addressing nutritional deficiencies, and supporting overall quality of life [19].

However, studies specific to the Kuwaiti population are limited, creating a significant gap in understanding the local context and needs. Currently, there are no data to describe the nutritional status of pediatric patients with CD in Kuwait and the associated demographic and socioeconomic factors. Addressing this gap is essential for improving patient care plans and developing effective intervention strategies to tackle malnutrition and growth issues in this vulnerable population.

Therefore, this study aimed to assess the nutritional status of children with CD in Kuwait using anthropometric measurements and biochemical parameters, and to identify the nutritional deficiencies and sociodemographic factors associated with growth stunting in these patients. By doing so, the study seeks to provide valuable insights that can inform healthcare practices and policies to enhance the management and support of children with CD in Kuwait.

## 2. Materials and Methods

### 2.1. Study Design and Population

This case–control descriptive study was conducted between November 2017 and December 2019. Between April 2017 and December 2019, 548 children aged 3–18 years were registered as case referrals to outpatient pediatrics gastroenterology clinics in all included hospitals. Patients with celiac disease (CD) were invited to participate in the study during a multicenter recruitment process conducted at outpatient pediatric gastroenterology clinics in hospitals under the jurisdiction of the Ministry of Health (MOH) in Kuwait. The participating hospitals included Al-Amiri, Al-Sabah, Al-Adan, and Mubarak Al-Kabeer hospitals. The participants in the CD group were aged 3–18 years. We used the ESPGHAN criteria for diagnosing CD, including IgA anti-tissue transglutaminase antibodies (ATTGa) and duodenal biopsy, a protocol recommended for this region [20]. The duodenal biopsy is not mandatory if antibody levels exceed the upper normal range by ten times [21]; however, none of the patients reached this level. Therefore, we excluded children with positive serological test results who refused duodenal biopsy. We also excluded those who were participating in any other ongoing interventional study and those with CD combined with Down syndrome or Turner syndrome.

Thirty healthy children from pediatric clinics at primary care centers were assigned to the control group. These children were selected to ensure they did not have any chronic illnesses, including heart disease, other gastrointestinal diseases, diabetes, renal disease, or cancer, and they had negative serological test results. Ethical approval was obtained from the MOH (approval number: 2018/819), and written informed consent was obtained from the parents of all participating children. Participation was voluntary and did not affect the patients’ rights to treatment.

#### Justification for Choice of Controls

In this study, we selected healthy children without any chronic illnesses, including heart disease, other gastrointestinal diseases, diabetes, renal disease, or cancer, and those with negative serological test results, to serve as the control group. To ensure comparability and reduce potential confounding, we used sex- and age-matched controls. This decision was based on the need to have a comparable group that did not have CD or other conditions that could confound the results related to nutritional status. The controls were recruited from pediatric clinics at primary care centers, ensuring they represented a similar age range and geographic distribution as the case group. This approach helped reduce potential biases related to environmental and socioeconomic factors, as both groups were likely to have similar access to healthcare services and nutritional resources. By selecting healthy sex- and age-matched controls, we aimed to isolate the effects of CD on nutritional status, providing a clearer comparison and understanding of the specific nutritional deficiencies associated with CD.

### 2.2. Study Questionnaire

A survey questionnaire comprising 79 items was designed and tested initially on a random sample of patients (n = 15) to verify its clarity, consistency, redundancy, and reliability. The questionnaire comprised the demographic information of the child and family and the patient’s clinical data, medical history, and biochemical tests. The participants’ parents were interviewed by a pediatric dietitian who was a member of the research team. After conducting the pilot study, we reviewed the feedback and found that no further changes to the questionnaire were necessary.

### 2.3. Nutritional Assessment

#### 2.3.1. Anthropometric Measurements

The current weight of the children (kg) was measured using an electronic scale, with each patient wearing minimal clothing. Height was measured using a wall-mounted Harpenden stadiometer to the nearest centimeter. Anthropometric data were plotted on growth charts derived from the Centers for Disease Control and Prevention (CDC) using the 2020 QxMD Software Inc [20]. Height-for-age (H/A) and body mass index (BMI)-for-age curves were converted into percentiles and Z-scores to identify potential health- or nutrition-related problems, including growth stunting, wasting, and obesity. An H/A < 5th percentile indicated growth stunting (chronic undernutrition), whereas that >95th percentile indicated tall stature. BMI-for-age < 5th percentile indicated underweight (acute undernutrition), that from the 5th to <85th percentile was considered normal, that from the 85th to <95th percentile indicated overweight, and that ≥95th percentile indicated obesity. Moreover, a cross-classification scheme based on the adapted Water Low Classification (AWC) [21], involving the categories obtained by applying ±2 standard deviations (SDs) to the H/A Z-score (HAZ) and BMI-for-age, was applied. Based on the combination of the two categories obtained with HAZ (<−2 and >2 SD) and the three related to BMI-for-age (<−2, −2 to 2, and >2 SD), the children were classified according to six nutritional conditions with their established categories as follows: eutrophic, wasting/acute undernutrition, growth stunting/chronic undernutrition, overweight, concurrent growth stunting and wasting, and growth stunting plus overweight status. Thus, these indices illustrate the coexistence of more than one indicator of the child’s nutritional status [21].

#### 2.3.2. Adherence to GFD

The MOH provides several gluten-free (GF) products to each patient diagnosed with CD. Participants and their parents were asked whether they received GF products from the MOH to assess the availability of GF products for home meals. We also used the follow-up data on serum ATTGa levels to assess the current serology status of children who adhered to a GFD. Levels < 10 U/mL indicated good adherence to the GFD.

#### 2.3.3. Biochemical Tests

Biochemical tests, including tests for complete blood count (CBC) and ferritin, vitamin B12, and vitamin D levels, were performed for all participants. IDA was defined by a hemoglobin concentration < 115 g/L for participants aged 5–11 years, <120 g/L for those aged 12–14 years, and <130 g/L for those aged >15 years, according to the recommendations from the World Health Organization (WHO) [22]. Moreover, ferritin is an indicator of the body’s iron reserve, and serum ferritin levels < 15 μg/L suggest IDA [23]. The reference range of vitamin B12 level is 180–914 ng/L, but a serum vitamin B12 level < 150 ng/mL indicates vitamin B12 deficiency [24]. Serum 25-hydroxyvitamin D [25(OH)D] levels were measured using an electrochemiluminescence binding assay and an ECLIA Cobas e601 analyzer (Roche Diagnostics, Indianapolis, IN, USA). The reference ranges of serum 25(OH)D concentrations are as follows: <50 mmol/L, deficiency; 50–75 mmol/L, insufficiency; and >75 mmol/L, sufficiency [25].

### 2.4. Statistical Analysis

Statistical analysis was performed using IBM SPSS ^®^ Statistics version 24.0 (Somers, NY, USA). The Kolmogorov–Smirnov test was used to assess the normality of the continuous variables; the data were not normally distributed. Accordingly, continuous and categorical variables are presented as median (interquartile range [IQR], 25−75 percentile) and percentages, respectively. Differences between the categorical binary groups, such as comparisons between patients with CD and controls and between patients with CD with and without growth stunting, were analyzed using the Mann–Whitney U or chi-square test. Univariate logistic regression analyses were conducted to investigate the association between demographic and nutrition-related characteristics and CD to select the covariates that can be adjusted for the multivariate-adjusted logistic regression models. Two sets of multivariate logistic regressions were conducted. For the first set (1), the dependent variable (celiac group = 1 and non-celiac = 0) with a forward binary logistic regression analysis method was used to test the association with the following independent variables: age group, nationality, sex, mother’s educational attainment, father’s educational attainment, monthly family income, anemia categories, and serum vitamin D levels. For the second set (2), the dependent variable (stunted = 1; non-stunted = 0) with a forward binary logistic regression analysis was used, and the variable(s) entered in step 1 are as follows: age group; nationality; sex; mother’s educational attainment; father’s educational attainment; monthly family income; anemia categories; and serum vitamin D, ferritin, vitamin B12, and ATTGa levels. A *p*-value < 0.05 was considered statistically significant.

### 2.5. Sample Size Calculations

The sample size for our case–control study was determined based on the prevalence of anemia among CD patients (cases) at 30% and controls at 7%, using a significance level of 0.05 and a desired power of 80%. The optimal sample size calculation, considering these parameters, suggested the need for 100 cases and 100 controls to achieve the desired statistical power. However, due to recruitment limitations, we enrolled 77 cases and 30 controls. The power calculation was performed using the formula for comparing two proportions (Kelsey et al., 1996) [26]. The final power of the study, based on the actual sample sizes, was approximately 71%.

## 3. Results

Among the 548 children registered as case referrals to the outpatient pediatrics gastroenterology clinics in all included hospitals, 139 tested positive for CD serology. A total of 18 children were excluded: 11 refused duodenal biopsy and 7 had normal duodenal histopathology, resulting in a total of 121 confirmed CD patients with positive serology and duodenal biopsy. Of these 121 patients, 44 were further excluded: 6 due to having Down syndrome, and 38 either refused participation or were lost to follow-up. Moreover, 11 patients were excluded because of incomplete questionnaires, leaving 77 patients with CD eligible for inclusion in the study. Among the patients in this study, 14.3% (n = 11) had first-degree relatives diagnosed with CD, and 13% (n = 10) had second-degree relatives diagnosed with CD. Among healthy controls, 30 children met the inclusion criteria and were invited to participate in the study with their parents.

### 3.1. Demographic Characteristics

Of the included patients, 54.5% were girls, and 45.5% were boys (*p* = 0.672). Most participants were Kuwaiti nationals (Table 1). Non-Kuwaitis were from neighboring Arab countries, including Egypt (22.1%), Syria (11.7%), Jordan (2.6%), and Yemen (6.5%). The average age of participants was 10.3 years (SD = 3.71), with six children (7.8%) aged <5 years in the CD group. As for socioeconomic status, the families of 26% of participants with CD belonged to the low monthly income category (KD < 500–1000). None of the families of participants in the control group were in the low monthly income category; they mostly belonged to the high family monthly income category (KD > 2000) (20.8% vs. 63.3%, *p* = 0.0001). As for parents’ educational attainment, approximately a third of the mothers of patients with CD had secondary school or lower educational levels compared to those of mothers of the healthy controls (32.2% vs. 0%). The same was true for the fathers of patients with CD compared to those of controls (31.2% vs. 6.7%, *p* = 0.026).

### 3.2. Clinical Characteristics

Gastrointestinal symptoms, including constipation (39.0%), diarrhea (32.5%), bloating (42.9%), and abdominal pain (49.4%), were observed in 84.4% of patients with CD (Table 2). Itchy skin lesions were the least frequently reported symptom. Patients with CD experienced a high proportion of comorbidities, including type 1 diabetes mellitus (27.3%) and anemia (19.5%). Additionally, 41.6% of patients with CD reported taking medications, including insulin, macrogol with electrolytes (Movicol), ranitidine (Zantac), and esomeprazole (Nexium). Reports of weight loss were confirmed by the parents of 33.8% of patients with CD; however, 26% reported weight gain.

### 3.3. Nutritional Status: Anthropometric Assessment

Approximately one-third (31%) of the children with CD had growth stunting (H/A percentile < 5%), whereas there was no growth stunting in the healthy control group (Table 3). Based on BMI-for-age categories, there were more underweight participants (<5th percentile) among the children with CD than among healthy controls (20.8% vs. 3.3%; *p* = 0.026). More participants among children with CD were overweight and obese than those among healthy controls (6.5% vs. 0% and 27.3% vs. 20.1%, respectively; *p* = 0.026). When classifying the nutritional status of patients with CD according to AWC combined with anthropometric measurements (Table 3), chronic undernutrition/growth stunting (only), acute undernutrition/wasting (only), and concurrent growth stunting and wasting was observed in 22.1%, 15.6%, and 5.2% of patients, respectively. The occurrence of overweight status (only) was observed in 29.9% of patients, and growth stunting plus overweight status was observed in 3.9%. Normal nutritional status (eutrophic) was observed in 23.4% of patients. Among the healthy controls, 76.7% were eutrophic, 20% were overweight, and 3.3% exhibited wasting.

### 3.4. GFD Compliance

More than half of the patients with CD (63.6%) received GF products from the MOH, and 66.2% frequently visited a dietitian and complied with the GFD (Table 3). A confirmed negative serum ATTGa test result was found in 61% of patients with CD, whereas 39% continued to exhibit a positive result for the ATTGa test.

### 3.5. Biochemical Assessment

Vitamin B12 deficiency was found only in patients with CD, and 46.7% of patients were deficient. The incidence of IDA was significantly higher among patients with CD than among healthy controls (36.4% vs. 6.7%; *p* = 0.007). Low serum ferritin level was found in 40.1% of patients with CD, indicating IDA. Vitamin D deficiency was mainly detected among patients with CD (65.3% vs. 26.7%; *p* < 0.001), with the median concentration of serum vitamin D in children with CD being significantly lower than that in the control group (33.0 vs. 67.50; *p* = 0.001; Table 3 and Table 4).

### 3.6. Factors Associated with CD and Growth Stunting

Based on the univariate logistic regression analysis (Table 3), nationality, educational attainment of both parents, socioeconomic status, anemia, and vitamin D and vitamin B12 serum levels were significantly associated with the risk of CD. Higher odds of IDA (odds ratio [OR] = 8.00 [1.77–36.14]; *p* = 0.007) and vitamin D deficiency (OR = 6.65 [1.91–16.71], *p* = 0.002) were found in children with CD (Table 3). Lower serum vitamin D and B12 levels were significantly associated with CD (Table 4). As per a multivariate model in which all significant variables were fitted with forward logistic regression, lower socioeconomic status, anemia, decreased serum level of vitamin D, and younger age were factors significantly associated with a higher risk of CD (Table 5). As for growth stunting, lower educational attainment of both parents, non-Kuwaiti status, lower monthly family income, and lower serum ferritin levels were associated with a higher risk of growth stunting (Table 6). Based on the multivariate model fitted with forward logistic regression, lower maternal educational attainment, lower family income, and lower serum ferritin levels were independently associated with a higher risk of growth stunting (Table 7).

## 4. Discussion

To the best of our knowledge, this study is the first in Kuwait to provide detailed information on the nutritional status of children and adolescents with CD and related risk factors. Malnutrition, presented as growth stunting, was present in 30% of the patients with CD in this study, whereas low BMI-for-age was present in 20.8%. Some patients were identified to have both growth stunting and wasting (5.2%). The same findings were reported by several investigators, including Dehbozorgi et al., who showed that 31% and 29% of Iranian pediatric patients with CD had low body weight and low BMI-for-age, respectively [27]. Moreover, Setavand et al. found that the prevalence of short stature among 361 Iranian children with CD was 18.3%, according to the CDC criteria, and 10%, according to the WHO criteria; 20–30% of patients were malnourished and had a low BMI-for-age [28].

The prevalence of growth stunting in our study was much higher than that reported in Iran but lower than the 72% previously reported in Kuwait by Al-Qabandi et al. [6]. Interestingly, the Kuwait National Nutrition Survey data for school-aged children 2019 reported a prevalence of growth stunting of 3.70% [29]. None of the healthy controls in our study exhibited growth stunting. Surveys elsewhere have shown that children with short stature commonly have CD [30]. The risk of CD in patients with isolated stunted growth or short stature is estimated to be between 10% and 40% [7]. Masood et al. reported a 40% prevalence of CD among 300 children with short stature [31]. Among Saudi children, Assiri (2010) found CD to be a significant cause of short stature in children without gastrointestinal complaints [12]. Stunting is usually a form of long-term malnutrition accompanied by nutrient deficiencies, chronic infection, or disease. Growth failure, short stature, and stunting are the most common extraintestinal features of CD caused by nutrient malabsorption. However, the pathogenesis of CD-associated short stature remains unclear. Several proposed mechanisms have been considered, including nutrient deficiency, resistance to growth hormones, and low levels of IGF-1. Children with short stature usually present with reduced levels of IGF-1, IGF-2, and insulin-like growth factor binding protein (IGFBP)-3; increased levels of IGFBP-2 and IGFBP-1; and a blunted GH response to pharmacological stimuli [7]. A significant inverse association was found between the duration of gluten exposure and IGF-1 levels, and a substantial reduction in IGF-1 levels was observed after prolonged gluten exposure and before growth failure [7]. Gluten withdrawal from the diet is frequently associated with a marked improvement in linear growth within 2 years [32].

Growth stunting in childhood is associated with impaired fat oxidation, which is proposed as a mechanism involved in mediating obesity in at-risk populations [33]. This double burden of malnutrition increases the risks of mortality, morbidity, and poor cognitive development. Our data showed that growth stunting was concurrent with being overweight and obese in 3.9% of patients with CD. This phenomenon is also observed to a lesser extent among non-celiac school-aged children in Kuwait, among boys but not among girls [29]. Overweight and stunting have been found in transition countries, such as rural Mexico, Peru, Russia, Brazil, South Africa, and China [34,35,36,37]. In these countries, a diet of poor quality is typically low in animal protein, high in simple carbohydrates, and low in fat and micronutrients, which may inhibit linear growth while allowing for fat deposition and obesity [38]. In addition to poor dietary choices, fetal and infant undernutrition (especially during the first 1000 days of life), recurrent or chronic infections, and malabsorption conditions (such as CD) modulate the child’s nutritional status and growth [39,40].

In our CD group, 45% of patients had a normal BMI, whereas only 6.5% and 27.3% were overweight and obese, respectively. Our data are not different from those previously reported nationwide among children in Kuwait, with the prevalence of overweight and obesity in school-aged children being 20.19% and 28.39%, respectively [41]. There are several explanations for overweight and obesity in children with CD; however, few have a well-established association with CD [42,43]. Following treatment, increased well-being may increase food intake and daily activity. Alterations in diet due to dietary restrictions and close nutritional surveillance can affect the weight and BMI of patients with CD. Overweight and obesity are becoming more common in patients with CD than previously reported.

More than 60% of the patients with CD in this study were compliant with a GFD. Compared with those who were non-compliant, compliant patients had a trend for higher percent fat mass (27.9 [8.98] vs. 23.20 [8.79], *p* = 0.089) and a lower percentage of fat-free mass (72.37 [9.17] vs. 77.65 [9.19], *p* = 0.063; data not in tables). It was unclear whether their body composition was due to a GFD, as we did not track their weight status or body composition at diagnosis. In a US cohort study, Reilly et al. showed that the GFD has a beneficial effect on most children who were overweight with CD [44]. However, some children who were overweight had worsened BMI Z-scores while being compliant with a GFD, and non-compliant patients who were overweight showed an increased BMI at follow-up. Prospective studies are needed among our patients with CD in Kuwait to identify factors related to changes in body composition, including physical activity, dietary choices, daily total energy intake, and macronutrient intake.

Our data showed that children with CD were eight times more likely to have IDA (OR, 8.00 [1.771–36.135]; *p* = 0.007), and 36.4% had IDA. Notably, the prevalence of IDA (19.5%) was much higher than its reported value (Table 2), indicating a lack of awareness. These results are consistent with those of several studies on pediatric and adult populations [45,46]. In a multicenter Italian study that included 1026 patients with subclinical/silent CD, IDA was found in 46% of adults and 35% of pediatric patients [47]. Anemia in children with CD adversely affects motor, cognitive, and socioemotional development and alters neuromaturation [48]. It is a relatively frequent condition in CD caused by iron loss and reduced iron absorption, which might result from the reduced expression of different proteins regulating iron absorption. Even after adopting a GFD, anemia persists because a GFD often lacks high iron content. Notably, our data showed that adolescent girls had a higher prevalence of IDA than boys among both patients and controls, which might be partly explained by blood loss during menstruation and worse dietary habits compared to those of boys, as previously reported among Kuwaiti adolescents [49]. A good approach to prevent malnutrition and maintain growth in children with CD is to start a GFD supported by oral iron administration [45,47,50].

Vitamin B12 deficiency was found in 46.6% of patients with CD, and 15% had megaloblastic anemia. In our study, the prevalence of vitamin B12 deficiency in patients with CD was higher than that mentioned in other reports. Dickey et al. showed that circulating levels of vitamin B12 were inadequate in 5–40% of patients with CD at diagnosis and in 2.9–41% of patients following a GFD [51]. Although the absorption site of vitamin B12 is preserved in patients with CD, vitamin B12 deficiency is still common in this population [52]. Vitamin B12 deficiency in children with CD can lead to developmental delays, weakness, and FTT. Dietitians must promote dietary vitamin B12 by increasing the intake of fortified cereals, eggs, animal liver, and kidneys. Numerous studies recommend starting vitamin B12 supplementation to meet these requirements and protect against neurological complications [52]. Moreover, a long-term GFD is an effective treatment for normalizing vitamin B12 levels among patients with CD [53]. Moreover, frequent vitamin B12 screening may be beneficial for reducing the risk of deficiency-related diseases [54].

Furthermore, this study found vitamin D deficiency and insufficiency in 65.3% and 18.7% of patients, respectively. The patients with CD in our study were six times more likely to have vitamin D deficiency than the controls (OR, 6.65 [1.91–16.71]; *p* = 0.002). Rotondi et al. found that vitamin D deficiency is a common feature, reaching up to 52% among children with CD at diagnosis [55]. Vitamin D deficiency is a critical health problem leading to muscle weakness, muscular pain, and rickets in children. Low sunshine exposure, skin pigmentation, air pollution, skin covering, and low vitamin D intake may be responsible for this deficiency; however, damage to the small intestinal mucosa, especially at diagnosis, may be a contributing factor. Decreased intestinal absorption of calcium and vitamin D may be due to chronic intestinal inflammation, which may lead to the release of proinflammatory cytokines with a subsequent increase in bone loss [52,55]. Therefore, children with CD on a GFD need vitamin D and calcium supplementation to bring levels within a normal range [52,55].

Our study revealed that a low socioeconomic status was associated with a higher risk of CD and worsening of anemia, low ferritin and vitamin D levels, and growth stunting, as shown in our multivariate logistic regression models. Many of our patients with CD were non-Kuwaitis (42.9% vs. 13.3%, *p* = 0.004). The non-Kuwaitis in this study were from low-income families, which may have contributed to a gap in health literacy and access to health information, similar to disparities observed in different socioeconomic brackets elsewhere [56]. Patients with CD who had a lower socioeconomic status and education level were less inclined to obtain GF products due to their cost.

Despite a high compliance rate with a GFD in our sample, many children with CD have vitamin D and B12 deficiencies and low hemoglobin and serum ferritin levels. Dietary adherence to a GFD is paramount as it is the only treatment available for CD. However, a GFD is insufficient to offset poor eating patterns in children with CD, which can lead to malnutrition, obesity, and related chronic diseases. Dietitians can play an essential role by following up with dietary adherence to a GFD, correcting nutritional deficiencies, and preventing the development of possible comorbidities in children with CD. Studies are needed to identify novel targets for therapy and lifestyle and behavioral recommendations to improve patient outcomes and support adherence to a GFD. Family-based intervention may be a workable strategy for educating patients with CD, supporting adherence to a GFD, improving nutritional status, and preventing nutritional deficiency.

Early identification of symptoms may help prevent the delayed diagnosis of CD, which causes severe malnutrition and growth impairment. Moreover, increasing awareness among healthcare professionals, including pediatricians and dietitians, on new clinical manifestations and nutritional deficiencies common among patients with CD, is essential to provide appropriate and efficacious monitoring strategies and interventions in future care plans. An annual review and follow-up after CD diagnosis, including vitamin D, B12, folate, CBC, and ferritin tests, should be performed to screen for possible emerging nutritional deficiencies [56]. Regular follow-up with a dietitian is important for developing healthy dietary habits that prevent malnutrition and obesity. Moreover, access to a dietitian may provide the best choices for GF products with high nutritional quality.

Nevertheless, this study has several limitations. The primary limitation is the small sample size of the control group compared to that of the case group. Additionally, we did not include participants accessing all health sectors, such as public and private clinics, limiting the generalizability of our findings to all children and adolescents with CD in Kuwait. This imbalance in recruitment could affect the findings related to parental socioeconomic status and educational attainment. We acknowledge several limitations due to our narrow inclusion criteria. These criteria, while ensuring a homogeneous study population, limit the generalizability of our findings to all children with CD. Excluding certain comorbid conditions, children participating in other studies, and those outside the 3–18 age range, may overlook important variations in nutritional status and disease manifestations. Furthermore, restricting the study to specific hospitals under the Ministry of Health in Kuwait may introduce geographic and socioeconomic biases, potentially skewing the results. Future research should aim to address the impact of socioeconomic disparities in families of children with CD by utilizing a larger and more representative sample and include a broader and more diverse population to enhance the generalizability and applicability of findings across different demographics and healthcare settings.

## 5. Conclusions

This study examined the nutritional status of children with CD in Kuwait using biochemical parameters and anthropometric measurements, emphasizing the need for comprehensive assessments to identify and prevent significant nutritional deficiencies and growth impairment. Our study shows that a significant proportion of children and adolescents with CD had malnutrition, overt deficiencies, and impaired growth despite adhering to a GFD. Regular follow-ups with a dietitian are important to track dietary habits, ensure GFD adherence, and correct nutritional deficiencies. Special attention should be paid to children from families with an economic disadvantage. Further studies are needed to identify novel targets for therapy and lifestyle and behavioral recommendations that can improve patient outcomes and support adherence to a GFD. Family-based intervention may be a workable strategy for educating patients and promoting adherence to a GFD.

## Figures and Tables

**Table 1:** Demographic characteristics of participants.

Characteristics; n (%)	Variable	Patients with CD (n = 77)	Healthy Controls (n = 30)	Univariate Logistic Regression
Sex	Female	42 (54.5)	15 (50.0)	*p* = 0.672
	Male	35 (45.5)	15 (50.0)	
Age group, years	<5	5 (6.5)	-	*p* = 0.418
	5–9	27 (35.1)	11 (36.7)	
	10–14	36 (46.8)	15 (50.0)	
	15–19	9 (11.7)	4 (13.3)	
Type of CD case	Follow-up	56 (72.7)	-	
	Newly diagnosed	21 (27.2)	-	
Nationality	Kuwaiti	44 (57.1)	26 (86.7)	OR, 4.875 (1.55–15.33); *p* = 0.007
	Non-Kuwaiti	33 (42.9)	4 (13.3)	
Participating hospital	Al-Amiri	45 (58.4)	11 (36.7)	*p* = 0.102
	Mubarak Al-Kabeer	14 (18.2)	5 (16.7)	
	Al-Adan	10 (13.0)	9 (30.0)	
	Al-Sabah	8 (10.4)	5 (16.7)	
Respondent	Mother	11 (14.3)	-	
	Father	3 (3.9)	-	
	Both	60 (77.9)	30 (100)	
	Others	3 (3.9)	-	
Educational attainment, mother	Less than Secondary	9 (11.7)	-	OR, 0.442 (0.245–0.798); *p* = 0.007
	Secondary School	16 (20.8)	-	
	Diploma	18 (23.4)	12 (40)	
	BSc and above	34 (44.2)	18 (60)	
Educational attainment, father	Less than Secondary	8 (10.4)	-	OR, 0.556 (0.327–0.945); *p* = 0.030
	Secondary School	16 (20.8)	2 (6.7)	
	Diploma	16 (20.8)	10 (38.5)	
	BSc and above	37 (48.1)	18 (32.7)	
Socioeconomic status (total monthly family income)	KD < 500	7 (9.1)	-	OR, 0.339 (0.202–0.571); *p* < 0.001
	KD 500–1000	13 (16.9)	-	
	KD 1001–1500	25 (32.5)	5 (16.7)	
	KD 1501–2000	16 (20.8)	6 (20.0)	
	KD > 2000	16 (20.8)	19 (63.3)	

OR, odds ratio; CD, celiac disease; KD, Kuwaiti Dinar.

**Table 2:** Reported comorbid conditions and clinical symptoms in patients with celiac disease versus healthy controls.

Characteristics n (%)	Variable	Patients with Celiac Disease n = 77	Healthy Controls n = 30	*p*-Value
Reported Symptoms	Yes	65 (84.4)	2 (6.7)	<0.001
	No	12 (15.6)	28 (93.3)	
Constipation	Yes	30 (39.0)	1 (3.3)	<0.001
	No	47 (61.0)	29 (96.7)	
Diarrhea	Yes	25 (32.5)	--	
	No	52 (67.5)	30 (100)	
Bloody stool	Yes	14 (18.2)	--	
	No	63 (81.8)	30 (100)	
Fatigue	Yes	12 (15.6)	--	
	No	65 (84.4)	30 (100)	
Vomiting	Yes	21 (27.3)	--	
	No	56 (72.7)	30 (100)	
Abdominal pain	Yes	38 (49.4)	--	
	No	39 (50.6)	30 (100)	
Bloating	Yes	33 (42.9)	1 (3.3)	<0.001
	No	44 (57.1)	29 (96.7)	
Itchy skin lesions	Yes	5 (6.5)	1 (3.3)	
	No	72 (93.5)	29 (96.7)	
T1DM ^a^	Yes	21 (27.3)	--	
	No	56 (72.7)	30 (100)	
Anemia ^b^	Yes	15 (19.5)	1 (3.3)	0.037
	No	62 (80.5)	29 (96.7)	
Hypothyroidism	Yes	2 (2.6%)	0 (0%)	
	No	75 (97.4%)	30 (100%)	
Medication	Yes	32 (41.6)	--	
	No	45 (58.4)	30 (100)	

^a.^ TIDM, type 1 diabetes mellitus; ^b.^ anemia physician—diagnosed based on hemoglobin concentrations.

**Table 3:** Participants’ nutrition-related characteristics in each category, n (%).

Category, n (%)		Patients with CD (n = 77)	Healthy Controls (n = 30)	Univariate Logistic Regression
Stature-for-age ^1^	Short stature	24 (31)	-	*p* = 0.997
	Normal	46 (59.7)	30 ^b^ (100)	
	Tall stature	7 (9.1)	-	
BMI-for-age ^2^	Underweight	16 ^a^ (20.8)	1 ^b^ (3.30)	*p* = 0.871
	Normal weight	35 ^a^ (45.5)	23 ^b^ (76.6)	
	Overweight	5 (6.5)	-	
	Obese	21 (27.3)	6 (20.1)	
Nutritional status ^3^	Undernutrition/growth stunting only	17 (22.1)	-	
	Undernutrition/wasting only	12 (15.6)	1 (3.3)	
	Concurrent stunting and wasting	4 (5.2)	-	
	Stunting and overweight	3 (3.9)	-	
	Overweight only	23 (29.9)	6 (20)	
	Normal eutrophic	18 (23.4)	23 (76.7)	
IDA	Yes	28 (36.4)	2 (6.7)	OR, 8.00 (1.771–36.135); *p* = 0.007
	No	49 (63.3)	28 (93.3)	
Iron deficiency	Low ferritin	30 (41.1)	-	*p* = 0.998
	Normal ferritin	43 (58.9)	30 (100)	
Vitamin D status	Deficiency	49 (65.3)	8 (26.7)	OR, 6.65 (1.91–16.71); *p* = 0.002
	Insufficiency	13 (17.3)	10 (33.3)	*p* = 0.754
	Normal	13 (17.3)	12 (40)	One ref
Vitamin B12 status	Deficiency	28 (46.7)	-	*p* = 0.996
	Megaloblastic anemia	9 (15)	-	
	Normal	23 (38.3)	30 (100)	

BMI, body mass index; IDA, iron-deficiency anemia; CD, celiac disease; OR, odds ratio. A *p*-value < 0.05 was considered statistically significant. ^1^ According to the CDC, stature-for-age was classified as follows: short stature, <5th percentile; normal stature, ≥5th to <95th percentile; and tall stature, ≥95th percentile. ^2^ The classifications of BMI are as follows: underweight, <5th percentile; normal weight, 5th to 85th percentile; overweight, ≥85th percentile; and obesity, ≥95th percentile [20]. ^3^ Adapted from Water Low Classification [21]. ^ab^ Different superscript letters indicated significantly different at *p* < 0.05.

**Table 4:** Anthropometric and biochemical parameters as continuous variables.

Characteristics	Patients with CD (n = 77)	Healthy Controls (n = 30)	*p*-Value	Mann–Whitney U Test	Univariate Logistic Regression
Height, cm	134.4 ± 19.3	145.0 ± 16.8	0.010		
	133 (122.75–149.30)	144.50 (130.75–160.25)		*X*^2^ = 4.490; df = 1; *p* = 0.034	OR = 0.969 (0.946–0.993); *p* = 0.013
Stature-for-age percentile	39.9 ± 36.4	60.4 ± 22.3	0.005		
	26.44 (3.84–76.96)	61.60 (47.01–79.27)		*X*^2^ = 8.170; df = 1; *p* = 0.004	OR = 0.982 (0.969–0.995); *p* = 0.007
Weight, kg	37.7 ± 22.5	42.2 ± 16.1	0.326		
	30.00 (23.00–45.00)	39.00 (29.23–52.750		*X*^2^ = 3.990; df = 1; *p* = 0.046	*p* = 0.326
Weight-for-age percentile	47.5 ± 39.2	64.2 ± 25.3	0.033		
	36.69 (8.69–91.92)	66.79 (47.71–85.44)		*X*^2^ = 5.895; df = 1; *p* = 0.015	OR = 0.987 (0.975–0.999); *p* = 0.036
BMI, kg/m^2^	19.4 ± 6.2	19.3 ± 3.8	0.981		
	50.10 (8.15–92.80)	18.75 (16.58–21.38)		*p* = 0.256	*p* = 0.981
BMI-for-age percentile	50.8 ± 38.9	61.5 ± 23.9	0.164		
	123.00 (113.00–131.00)	61.0 (44.85–79.48)		*p* = 0.480	*p* = 0.164
Hemoglobin	122.9 ± 12.9	129.6 ± 8.3	0.01		
	123.00 (113.00–131.00)	129.50 (124.25–135.00)		*p* = 0.091	OR = 0.943 (0.905–0.984); *p* = 0.006
Ferritin	35.5 ± 37.3	35.3 ± 18.8	0.985		
	21.00 (8.91–50.54)	30.0 (19.80–47.25)		*p* = 0.251	*p* = 0.998
Serum vitamin D	179 ± 101.5	288.8 ± 71.9	<0.001		
	33.00 (22.00–67.00)	67.50 (43.75–112.25)		*X*^2^ = 10.905; df = 1; *p* < 0.001	OR = 0.414 (0.245–0.701); *p* = 0.001
Serum vitamin B12	48.9 ± 39.3	80.2 ± 48.8	0.001		
	167.00 (100.25–224.00)	281.00 (237.75–333.50)		*X*^2^ = 26.450; df = 1; *p* < 0.001	OR = 0.988 (0.982–0.993); *p* < 0.001

Values are presented as medians (IQR). A *p*-value < 0.05 was considered statistically significant. BMI, body mass index; OR, odds ratio; CD, celiac disease; IQR, interquartile range; SD, standard deviation; *X*^2^, chi-squared; and df, degrees of freedom difference between medians; the Mann–Whitney U test was used.

**Table 5:** Multivariate logistic regression for the factors associated with the risk of celiac disease.

**Independent** **Variables**	**B**	**SE**	**Wald**	**df**	***p*-Value**	**Exp(B)**	**95% CI for Exp(B)**		R = 0.509
							**Lower**	**Upper**	
Age group	−1.26	0.47	7.33	1	0.007	0.28	0.11	0.71	
Monthly family income	−1.18	0.31	14.53	1	<0.001	0.31	0.17	0.57	
Anemia categories	2.69	0.93	8.48	1	0.004	14.76	2.41	90.36	
Serum vitamin D	−0.996	0.36	7.52	1	0.006	0.37	0.18	0.75	
Constant	7.54	2.24	11.36	1	<0.001	1880.11			

Dependent variable (celiac group = 1 and non-celiac = 0). Forward binary logistic regression analysis was used. The variables entered in step 1 are as follows: age group, nationality, sex, mother’s educational attainment, father’s educational attainment, monthly family income, anemia categories, and serum vitamin D levels. df, degrees of freedom; CI, confidence interval; B, beta unadjusted coefficient; and Wald test to determine statistical significance for each independent variable.

**Table 6:** Categorical characteristics of participants with CD with growth stunting (n = 24).

Categorical Characteristics		With Growth Stunting *, % (n)	*p*-Value
Mother’s education	Secondary school or less	14 (56.0)	<0.001
	College diploma and higher	10 (12.2)	
Father’s education	Secondary school or less	10 (38.5)	0.032
	College diploma and higher	14 (17.3)	
Nationality	Kuwaiti	8 (11.4)	<0.001
	Non-Kuwaiti	24 (22.4)	
Monthly family income	KD < 500	5 (71.4)	<0.001
	KD 500–1000	7 (53.8)	
	KD 1001–1500	8 (26.7)	
	KD 1501–2000	4 (18.2)	
	KD > 2000	0	
Weight status	Underweight	4 (23.5)	0.164
	Normal Weight	17 (29.3)	
	Overweight	1 (20.0)	
	Obese	2 (7.4)	
Vitamin D deficiency	Deficient	15 (26.3)	0.353
	Insufficient	5 (21.7)	
	Sufficient	3 (12.0)	
Anemia	Yes	8 (33.3)	0.339
	No	16 (66.7)	
Ferritin	<12 ng/mL (low)	14 (46.7)	<0.001
	12–306.8 ng/mL (normal)	9 (12.3)	
ATTGa	Negative (compliant)	14 (18.2)	0.122
	Positive (non-compliant)	10 (33.3)	
Vitamin B12	180–914 ng/mL (normal)	10 (18.9)	0.337
	150 to <180 ng/mL (megaloblastic anemia)	3 (33.3)	
	<150 ng/mL (deficiency)	9 (32.1)	

CD, celiac disease; ATTGa, IgA anti-tissue transglutaminase antibody. * Reference group: tall participants merged with normal-height participants to have a binary variable, with and without stunting.

**Table 7:** Multivariate logistic regression for the factors associated with stunting.

**Independent Variables**	**B**	**SE**	**Wald**	**df**	***p*-Value**	**Exp(B)**	**95% CI for Exp(B)**		R = 0.516
							**Lower**	**Upper**	
Mother’s education	−0.97	0.39	6.15	1	0.013	0.38	0.18	0.82	
Monthly family income	−0.78	0.34	5.24	1	0.022	0.46	0.24	0.89	
Ferritin	−1.49	0.68	4.76	1	0.029	0.23	0.06	0.86	
Constant	6.63	1.79	13.82	1	<0.001	760.33			

Dependent variable (stunted = 1; non-stunted = 0). Forward binary logistic regression analysis was used. The variable(s) entered in step 1 are as follows: age group, age at diagnosis, nationality, sex, mother’s education, father’s education, monthly family income, type 1 diabetes, anemia categories, serum vitamin D, ferritin, vitamin B12, and IgA anti-tissue transglutaminase antibody. SE, standard error; df, degrees of freedom; CI, confidence interval; B, beta unadjusted coefficients; Wald, a test-to-test significance for independent variables; and Exp(B), odds ratios.

## Data Availability

The raw data supporting the conclusions of this article will be made available by the authors on request.

## References

[1] Dunne M.R., Byrne G., Chirdo F.G., Feighery C. (2020). Coeliac disease pathogenesis: The uncertainties of a well-known immune-mediated disorder. Front. Immunol..

[2] Szajewska H., Shamir R., Mearin L., Ribes-Koninckx C., Catassi C., Domellöf M., Fewtrell M.S., Husby S., Papadopoulou A., Vandenplas Y. (2016). Gluten introduction and the risk of coeliac disease: A position paper by the European Society for Pediatric Gastroenterology, Hepatology, and Nutrition. J. Pediatr. Gastroenterol. Nutr..

[3] Farage P., Zandonadi R. (2014). The gluten-free diet: Difficulties celiac disease patients have to face daily. Austin J. Nutr. Food Sci..

[4] Rajani S., Alzaben A., Shirton L., Persad R., Huynh H.Q., Mager D.R., Turner J.M. (2014). Exploring anthropometric and laboratory differences in children of varying ethnicities with celiac disease. Can. J. Gastroenterol. Hepatol..

[5] Tortora R., Capone P., De Stefano G., Imperatore N., Gerbino N., Donetto S., Monaco V., Caporaso N., Rispo A. (2015). Metabolic syndrome in patients with coeliac disease on a gluten-free diet. Aliment. Pharmacol. Ther..

[6] Al-Qabandi W., Buhamrah E., Al-Abdulrazzaq D., Hamadi K., Al Refaee F. (2015). Celiac disease in children: Is it a problem in Kuwait?. Clin. Exp. Gastroenterol..

[7] Boguszewski M.C., Cardoso-Demartini A., Geiger Frey M.C., Celli A. (2015). Celiac disease, short stature and growth hormone deficiency. Transl. Gastrointest. Cancer.

[8] Weiss B., Skourikhin Y., Modan-Moses D., Broide E., Fradkin A., Bujanover Y. (2008). Is the adult height of patients with celiac disease influenced by delayed diagnosis?. Am. J. Gastroenterol..

[9] Kreutz J.M., Heynen L., Vreugdenhil A.C.E. (2023). Nutrient deficiencies in children with celiac disease during long term follow-up. Clin. Nutr..

[10] Moya D.A., Nugent C.A., Baker R.D., Baker S.S. (2020). Celiac Disease Nutritional Status and Poor Adherence to Follow-up. Clin. Pediatr..

[11] Allowaymi S.S., Binobead M.A., Alshammari G.M., Alrasheed A., Mohammed M.A., Yahya M.A. (2022). Nutritional Status of Saudi Children with Celiac Disease Following the Ministry of Health’s Gluten-Free Diet Program. Nutrients.

[12] Assiri A.M. (2010). Isolated short stature as a presentation of celiac disease in Saudi children. Pediatr. Rep..

[13] Husby S., Koletzko S., Korponay-Szabó I.R., Mearin M.L., Phillips A., Shamir R., Troncone R., Giersiepen K., Branski D., Catassi C. (2012). European Society for Pediatric Gastroenterology, Hepatology and Nutrition guidelines for the diagnosis of coeliac disease. J. Pediatr. Gastroenterol. Nutr..

[14] Mędza A., Szlagatys-Sidorkiewicz A. (2023). Nutritional Status and Metabolism in Celiac Disease: Narrative Review. J. Clin. Med..

[15] Pinto-Sanchez M.I., Blom J.J., Gibson P.R., Armstrong D. (2024). Nutrition Assessment and Management in Celiac Disease. Gastroenterology.

[16] Cheng F.W., Handu D. (2020). Nutrition Assessment, Interventions, and Monitoring for Patients with Celiac Disease: An Evidence Analysis Center Scoping Review. J. Acad. Nutr. Diet..

[17] Abdi F., Zuberi S., Blom J.J., Armstrong D., Pinto-Sanchez M.I. (2023). Nutritional Considerations in Celiac Disease and Non-Celiac Gluten/Wheat Sensitivity. Nutrients.

[18] Pinto-Sanchez M.I., Bai J.C. (2019). Toward New Paradigms in the Follow Up of Adult Patients With Celiac Disease on a Gluten-Free Diet. Front. Nutr..

[19] Dennis M., Lee A.R., McCarthy T. (2019). Nutritional Considerations of the Gluten-Free Diet. Gastroenterol. Clin. N. Am..

[20] Kuczmarski R.J., Kuczmarski M.F., Roche A.F. (2002). 2000 CDC growth charts. Top. Clin. Nutr..

[21] Ferreira H.D.S. (2020). Anthropometric assessment of children’s nutritional status: A new approach based on an adaptation of Waterlow’s classification. BMC Pediatr..

[22] World Health Organization Hemoglobin Concentrations for the Diagnosis of Anemia and Assessment of Severity. https://www.who.int/publications/i/item/WHO-NMH-NHD-MNM-11.1.

[23] World Health Organization Serum Ferritin Concentrations for the Assessment of Iron Status and Iron Deficiency in Populations. https://www.who.int/publications/i/item/9789240008526.

[24] De Benoist B. (2008). Conclusions of a WHO technical consultation on folate and vitamin B12 deficiencies. Food Nutr. Bull..

[25] Institute of Medicine (IOM) (2011). Dietary Reference Intakes for Calcium and Vitamin D.

[26] Kelsey J.L., Whittemore A.S., Evans A.S., Thompson W.D. (1996). Methods in Observational Epidemiology.

[27] Dehbozorgi M., Honar N., Ekramzadeh M., Saki F. (2020). Clinical manifestations and associated disorders in children with celiac disease in southern Iran. BMC Pediatr..

[28] Setavand Z., Ekramzadeh M., Honar N. (2021). Evaluation of malnutrition status and clinical indications in children with celiac disease: A cross-sectional study. BMC Pediatr..

[29] Al-Taiar A., Al Qaoud N., Sharaf Alddin R., Alanezi F., Subhakaran M., Dumadag A., Albatineh A.N. (2021). Stunting and combined overweight with stunting among school children in Kuwait: Trends over 13 years. Med. Princ. Pract..

[30] Singh A.D., Singh P., Farooqui N., Strand T., Ahuja V., Makharia G.K. (2021). Prevalence of celiac disease in patients with short stature: A systematic review and meta-analysis. J. Gastroenterol. Hepatol..

[31] Masood J., Rehman H., Anjum Z.M., Iqbal I., Zafar S., Ayesha H. (2020). Prevalence of celiac disease in idiopathic short stature children presenting in OPD of children hospital, Faisalabad. Ann. Punjab. Coll..

[32] Kozioł-Kozakowska A., Salamon D., Grzenda-Adamek Z., Krawczyk A., Duplaga M., Gosiewski T., Kowalska-Duplaga K. (2021). Changes in diet and anthropometric parameters in children and adolescents with celiac disease-one year of follow-up. Nutrients.

[33] Hoffman D.J., Sawaya A.L., Verreschi I., Tucker K.L., Roberts S.B. (2000). Why are nutritionally stunted children at increased risk of obesity? Studies of metabolic rate and fat oxidation in shantytown children from São Paulo, Brazil. Am. J. Clin. Nutr..

[34] Popkin B.M., Richards M.K., Montiero C.A. (1996). Stunting is associated with overweight in children of four nations that are undergoing the nutrition transition. J. Nutr..

[35] Fernald L.C., Neufeld L.M. (2007). Overweight with concurrent stunting in very young children from rural Mexico: Prevalence and associated factors. Eur. J. Clin. Nutr..

[36] Bain L.E., Awah P.K., Geraldine N., Kindong N.P., Sigal Y., Bernard N., Tanjeko A.T. (2013). Malnutrition in Sub-Saharan Africa: Burden, causes and prospects. Pan. Afr. Med. J..

[37] Atsu B.K., Guure C., Laar A.K. (2017). Determinants of overweight with concurrent stunting among Ghanaian children. BMC Pediatr..

[38] Pettifor J.M. (2006). Combined stunting and overweight in young children—A paradox?. S. Afr. J. Clin. Nutr..

[39] Sunguya B.F., Ong K.I., Dhakal S., Mlunde L.B., Shibanuma A., Yasuoka J., Jimba M. (2014). Strong nutrition governance is a key to addressing nutrition transition in low and middle-income countries: A review of countries’ nutrition policies. Nutr. J..

[40] Al-Taiar A., Alqaoud N., Ziyab A.H., Alanezi F., Subhakaran M., Sharaf Alddin R., Anna Jeng H., Akpinar-Elci M. (2021). Time trends of overweight and obesity among schoolchildren in Kuwait over a 13-year period (2007–2019): Repeated cross-sectional study. Public Health Nutr..

[41] Dibaise J.K., Frank D.N., Mathur R. (2012). Impact of the gut microbiota on the development of obesity: Current concepts. Am. J. Gastroenterol. Suppl..

[42] Baothman O.A., Zamzami M.A., Taher I., Abubaker J., Abu-Farha M. (2016). The role of gut microbiota in the development of obesity and diabetes. Lipids Health Dis..

[43] Reilly N.R., Aguilar K., Hassid B.G., Cheng J., Defelice A.R., Kazlow P., Bhagat G., Green P.H. (2011). Celiac disease in normal-weight and overweight children: Clinical features and growth outcomes following a gluten-free diet. J. Pediatr. Gastroenterol. Nutr..

[44] Freeman H.J. (2015). Iron deficiency anemia in celiac disease. World J. Gastroenterol..

[45] Mazza G.A., Marrazzo S., Gangemi P., Battaglia E., Giancotti L., Miniero R. (2019). Oral iron absorption test with ferrous bis-glycinate chelate in children with celiac disease. Minerva Pediatr..

[46] Talarico V., Giancotti L., Mazza G.A., Miniero R., Bertini M. (2021). Iron deficiency anemia in celiac disease. Nutrients.

[47] Stefanelli G., Viscido A., Longo S., Magistroni M., Latella G. (2020). Persistent iron deficiency anemia in patients with celiac disease despite a gluten-free diet. Nutrients.

[48] Alkazemi D., Rahman A., Habra B. (2021). Alterations in glutathione redox homeostasis among adolescents with obesity and anemia. Sci. Rep..

[49] Sanseviero M.T., Mazza G.A., Pullano M.N., Oliveiro A.C., Altomare F., Pedrelli L., Dattilo B., Miniero R., Meloni G., Giancotti L. (2016). Iron deficiency anemia in newly diagnosed celiac disease in children. Minerva Pediatr..

[50] Dickey W., Kearney N. (2006). Overweight in celiac disease: Prevalence, clinical characteristics, and effect of a gluten-free diet. Am. J. Gastroenterol..

[51] Rondanelli M., Faliva M.A., Gasparri C., Peroni G., Naso M., Picciotto G., Riva A., Nichetti M., Infantino V., Alalwan T.A. (2019). Micronutrients dietary supplementation advices for celiac patients on a long-term gluten-free diet with good compliance: A review. Medicina.

[52] Caruso R., Pallone F., Stasi E., Romeo S., Monteleone G. (2013). Appropriate nutrient supplementation in celiac disease. Ann. Med..

[53] Friedman A. (2012). Micronutrient deficiencies in pediatric celiac disease. Infant Child Adolesc. Nutr..

[54] Rotondi Aufiero V., Fasano A., Mazzarella G. (2018). Non-celiac gluten sensitivity: How its gut immune activation and potential dietary management differ from celiac disease. Mol. Nutr. Food Res..

[55] Zanobini P., Lorini C., Lastrucci V., Minardi V., Possenti V., Masocco M., Garofalo G., Mereu G., Bonaccorsi G. (2021). Health literacy, socio-economic determinants, and healthy behaviours: Results from a large representative sample of Tuscany region, Italy. Int. J. Environ. Res. Public Health..

[56] Wessels M.M., van Veen I.I., Vriezinga S.L., Putter H., Rings E.H., Mearin M.L. (2016). Complementary serologic investigations in children with celiac disease is unnecessary during follow-up. J. Pediatr..

